# Phosphate in the Context of Cognitive Impairment and Other Neurological Disorders Occurrence in Chronic Kidney Disease

**DOI:** 10.3390/ijms23137362

**Published:** 2022-07-01

**Authors:** Merita Rroji, Andreja Figurek, Davide Viggiano, Giovambattista Capasso, Goce Spasovski

**Affiliations:** 1 Department of Nephrology, Faculty of Medicine, University of Medicine Tirana, 1001 Tirana, Albania; 2 Department of Internal Medicine, Medical Faculty, University of Banja Luka, 78000 Banja Luka, Bosnia and Herzegovina; andrejafigurek@yahoo.com; 3 Institute of Anatomy, University of Zurich, 8057 Zurich, Switzerland; 4 Department of Translational Medical Sciences, University of Campania “L. Vanvitelli”, 80138 Naples, Italy; davide.viggiano@unicampania.it (D.V.); gb.capasso@unicampania.it (G.C.); 5 BioGeM, Institute of Molecular Biology and Genetics, 83031 Ariano Irpino, Italy; 6 University Clinic for Nephrology, Medical Faculty, University St. Cyril and Methodius, 1000 Skopje, North Macedonia; spasovski.goce@gmail.com

**Keywords:** chronic kidney disease, phosphate, PTH, FGF23, nervous system, dementia

## Abstract

The nervous system and the kidneys are linked under physiological states to maintain normal body homeostasis. In chronic kidney disease (CKD), damaged kidneys can impair the central nervous system, including cerebrovascular disease and cognitive impairment (CI). Recently, kidney disease has been proposed as a new modifiable risk factor for dementia. It is reported that uremic toxins may have direct neurotoxic (astrocyte activation and neuronal death) and/or indirect action through vascular effects (cerebral endothelial dysfunction, calcification, and inflammation). This review summarizes the evidence from research investigating the pathophysiological effects of phosphate toxicity in the nervous system, raising the question of whether the control of hyperphosphatemia in CKD would lower patients’ risk of developing cognitive impairment and dementia.

## 1. Introduction

At least in Italy, old ladies encourage the children to eat fish because it is “rich in phosphorous and phosphorous is good for the brain”. This “folk science” piece was started in 1719 by Johann Thomas Hensing, who first described the high content of phosphorous in the brain. Because Brandt, in 1669, showed that phosphorous could glow in the dark, Georges Cabanis (1757–1808) suggested a relation between phosphorous and mental states. Therefore, in 1852 Jacob Moleschott asserted that “without phosphorous, there are no thoughts”. At the end of the 1800s, the brain’s phosphorous was found mainly in phospholipids (the brain is very rich in lipids). Jean-Baptiste André Dumas (1800–1884), the mentor of Pasteur, found that fish are particularly rich in phosphorous. Finally, the famous Harvard professor Jean Louis Rodolphe Agassiz (1807–1873), renowned for the glacial era theory, merged these ideas and suggested that eating fish was helpful in the brain. Twain, in 1871, quotes Agassiz: “Yes, Agassiz does recommend authors to eat fish, because the phosphorous in it makes brains. So far, you are correct. However, I cannot help you with a decision about the amount you need to eat—at least, not with certainty. If the specimen composition you send is about your usual fair average, I should judge that maybe a couple of whales would be all you would like for the present. Not the largest kind, but simply good middling-sized whales”. The subsequent manuscript takes some distance from this position and does not support the suggestion of eating whales.

### Kidney and Nervous System in CKD

The nervous system and kidneys are linked under a physiological state to maintain normal body homeostasis. In chronic kidney disease (CKD), damaged kidneys can impair the central nervous system (CNS), including cerebrovascular disease and cognitive impairment (CI) [1]. CKD is a considerable global health problem with a prevalence of about 10% of the world population. With its silent development and progression, it is difficult to diagnose the disease at its early stages to stop further progression. Patients with CKD have a higher cardiovascular risk, so many do not survive until ensued end-stage renal disease (ESRD).

Recently, kidney disease has been proposed as a new modifiable risk factor for dementia [2]. It is reported that uremic toxins may have direct neurotoxic (astrocyte activation and neuronal death) and/or indirect action through vascular effects (cerebral endothelial dysfunction, calcification, and inflammation) [3,4]. Due to the endothelial dysfunction, small-vessel pathology has been used to clarify the link between kidney and cerebral microvasculature that share similar anatomical and physiologic features.

The prevalence of clinically evident stroke and subclinical cerebrovascular disease (e.g., white matter hyperintensities) was higher in CKD when compared to the general population. A stroke is a cerebrovascular event that happens due to the interruption of blood flow into the brain resulting in neuronal death [5], where vascular disease is a notable cause of dementia. It is reported that the annual incidence of stroke is around 15.1% in hemodialysis, and 9.6% in CKD patients as third most common cardiovascular cause of death [6]. Conversely, the annual incidence of stroke in patients without CKD is reported as 2.6%.

## 2. Epidemiology and Etiopathogenesis of Cognitive Impairment in CKD

Besides other comorbidities and CKD complications, cognitive impairment is often seen in patients suffering from CKD and moreover, in those with end-stage renal disease. About 20–50% of patients with moderate CKD have CI or dementia [7,8]. It is evaluated that the prevalence of CI in hemodialysis patients is 30–60% [9,10]. However, the real prevalence is difficult to judge, as the CI assessment is not standardized yet. The Mini-Mental State Examination is used in most studies but with limited sensitivity. This issue is reported by the study performed by Murray et al., where further neuropsychological testing increased the previously diagnosed CI of 3% in hemodialysis patients with ages of 55 years and older to 87% [11]. A cross-sectional study of 613 Chinese hemodialysis patients with a mean age of 64 years showed that 37.2% had mild and 43.7% had substantial CI. Factors associated with significant CI were age, education level, history of stroke and hypertension, dialysis vintage, and single-pool Kt/V [12]. In the same study, serum phosphate levels did not significantly differ from patients without CI. 

The pathogenesis of CI in CKD is complex, and several hypotheses are in use to explain it [13]. The vascular hypothesis presents CI as a consequence of impaired brain hemodynamics [14], presented either as a stroke or subclinical vascular disease (white matter lesions, silent brain infarcts, microbleeds) [9]. These changes are related to traditional risk factors, as aging, diabetes mellitus, hypertension, hypercholesterolemia, and non-traditional risk factors, chronic inflammation, metabolic disorders, hypercoagulable state, and oxidative stress [9], all are frequently presented in CKD patients. Due to the decreased excretion in CKD, serum phosphate is elevated, and vitamin D insufficiently activated with a consequent parathyroid hormone hypersecretion (secondary hyperparathyroidism) that in turn leads to an increased calcium–phosphate product and vascular calcification development. 

Recent studies underlined the association of higher serum phosphate with incident dementia in a large cohort of 744,235 veterans [15]. This association was more pronounced in patients less than 60 years old. The exact mechanism on how hyperphosphatemia causes CI and dementia is not entirely clear. However, it is well established that higher serum phosphate is associated with an increased cardiovascular risk and mortality [16] and increased carotid intima–media thickness [17]. Subclinical atherosclerosis is associated with impaired cognitive function and atherosclerotic events with significant cognitive impairment [18]. Lastly, a current report that showed that high serum P and calcium–phosphate product are potential risk factors for cerebral small vessel disease of all subtypes, including lacunes, white matter hyperintensities, and cerebral microbleeds [19]. 

Interestingly, experimental studies demonstrated that hyperphosphatemia increased the production of reactive oxygen species, as well as increased oxidative stress and inflammatory response, thus causing endothelial dysfunction [20]. Hyperphosphatemia might be the link between vascular and neurodegenerative hypothesis, as the later emphasizes the importance of uremic toxins in cerebral endothelial dysfunction development. In addition, phosphate toxicity was reported to accelerate the mammalian aging process by imposing tissue injury and reducing survival [21]. Nevertheless, the association between hyperphosphatemia and CI is not yet thoroughly investigated. A prospective study that included 5529 older men in the Osteoporotic Fractures in Men (MrOS) study showed that higher serum phosphate was associated with a higher likelihood of poor executive function, but without an association with impaired global cognitive function or decline in executive or global cognition [22]. However, it is necessary to note that the participants in this study had well-preserved kidney function. Furthermore, a recent meta-analysis on five studies with 27,805 patients indicated a 35% increased risk of CI or dementia in patients with albuminuria [23,24].

Intact FGF-23 was also not associated with baseline cognitive function or incident cognitive impairment in the cohort of well-functioning older adults with a mean follow-up time of 5.8 years [25]. However, in patients with CKD, the Mini-Mental State Examination and Montreal cognitive assessment scores showed significantly negative correlation with serum phosphate and blood urea, serum creatinine, serum uric acid, serum potassium, and CKD stage [26]. In addition, the Salford kidney cohort study in which the Montreal Cognitive Assessment and Trail Making Test were used showed only a trend of higher phosphate levels in CKD patients that developed CI [27]. In this context, CKD has emerged as a potential risk factor for cognitive decline, future stroke, and subclinical vascular diseases such as cerebral small vessel disease [28]. It has been shown that elevated phosphate in patients with CKD causes arterial media calcification, endothelial dysfunction, and increased VCAM-1/ICAM-1 expression in endothelial cells [29], thus inducing vascular damage.

In patients with CKD stages III to V, the relation between kidney and cognitive function was evaluated independently of the vascular risk factors. Among the other factors, the retention of uremic toxins, especially phosphate, has been again presented as a CKD-specific factor, accountable for structural and functional cerebral changes in patients with CKD [3,29]. Finally, the high phosphate level was associated with inflammation, stress, depression, and dementia, including Alzheimer’s disease [30]. 

This review summarizes the evidence from research investigating the pathophysiological effects of phosphate toxicity in the nervous system.

## 3. Phosphate Homeostasis in the Physiological Milieu and CKD

Phosphate is one of the crucial elements of the human body. It has a vital role in skeletal growth, energy, and metabolic functions in the body. In the form of inorganic phosphate, it is a macronutrient that is crucial to various cellular functions, including structure, energy production, metabolic pathways, and signaling. The vast majority (85%) of phosphate in the body is in the form of hydroxyapatite [Ca10(PO4)6(OH)2], found in the extracellular matrix of bone and teeth, serving as the main phosphate reservoir. The remaining phosphate is present mainly in the viscera and skeletal muscle, and only 0.1%, mainly as inorganic phosphate (Pi), is present in extracellular fluids [31]. About 30% of total cellular phosphate is stored in the ER (endoplasmic reticulum) and used in the phosphorylation of various proteins, whereas around 20% is present in the mitochondria. The remaining cellular phosphate is present in the nucleus, Golgi complex, and lysosomes [32].

Apart from diet, multiorgan crosstalk, hormones, and other factors, a complex system coordinates and controls phosphate, retaining its serum levels within a normal range. Maintaining normal serum phosphate levels relies on the absorption of dietary phosphate in the gut, reabsorption, phosphate excretion in the kidney, and phosphate flux between the extracellular and skeletal pools. 

The absorbed phosphate joins the extracellular fluid and moves in and out of the skeletal pool (approximately 3 mg/kg per day). Type III Na/Pi cotransporters, as well as PiT-1 and PiT-2, are ubiquitously represented and mediate cellular Pi homeostasis in all cells. Phosphate homeostasis is held by positive and negative feedback loops implicating the bone, intestine, kidney, and parathyroid gland [33]. 

The leading players in the regulation of phosphate involve a trio of hormones: parathyroid hormone (PTH), calcitriol, and fibroblast growth factor 23 (FGF23). Phosphate is freely filtered via the glomerulus and reabsorbed through the renal sodium/phosphate type 2 cotransporters NaPi-IIa (SLC34A1), NaPi-IIc (SLC34A3), and PiT-2 (SLC20A2), represented on the luminal side proximal tubular epithelial cells. 

FGF23 and PTH control phosphate reabsorption in the kidney. The dietary phosphate is absorbed in the gut via passive paracellular diffusion and by active cell-mediated transport of phosphate, mediated by the NaPi-2b cotransporter on the luminal side of the enterocyte [34]. Elevated serum phosphate levels induce the secretion of FGF23 and PTH, decreasing NaPi-2a and NaPi-2c expression in the proximal tubule with subsequent phosphaturia.

FGF23 is a potent phosphaturic glycoprotein secreted by osteoblasts and osteocytes, which express its endocrine hormone effect by binding to the fibroblastic growth receptor and coreceptor klotho. Whereas FGFRs are abundantly expressed throughout the body, tissue presentation of a Klotho is tightly handled and thus selects the effect of FGF23 on target organs [35]. In addition, the increase in FGF23 diminishes phosphate absorption in the gut by inhibiting NaPi-2b expression and suppressing circulating calcitriol. The phosphaturic effect associated with the decreased level of 1,25(OH)2D3 is a mechanism of FGF23 to control hyperphosphatemia [34,36]. Recently, there has been evidence that FGF23 could also directly affect calcium and sodium transport in the distal nephron [37,38]. PTH is a hormone secreted from the parathyroid gland in response to elevated serum Pi and decreased serum calcium and acts on the kidney to induce phosphaturia. It should be said that the kidney is the central organ that controls phosphate homeostasis. 

Phosphate toxicity is a well-described phenomenon in CKD [39]. During CKD progression, tubular phosphate reabsorption is remarkably reduced by the dual effect of a compensatory raised concentration of PTH and FGF23. Besides, FGF23 suppresses vitamin D activation and decreases PTH synthesis and secretion, the primary trigger of chronic kidney disease—mineral and bone disorders (CKD-MBD) [40]. 

Although FGF23 levels are elevated since its cofactor klotho declines in CKD, both can still maintain serum phosphate within the normal range in the early and intermediate stages of CKD. Elevated PTH levels are also expected in uremic patients much earlier than hyperphosphatemia. PTH stimulates calcitriol synthesis that further contributes to increased serum calcium [41]. In contrast with the physiologic state where FGF23 acts on the parathyroid gland by diminishing gene expression and secretion in CKD in the absence of Klotho, the parathyroid gland shows resistance to FGF23, enhancing PTH secretion. However, these compensatory mechanisms are ineffective as CKD progresses so that phosphate retention may occur, and hyperphosphatemia develops. 

It is essential to underline that Pi and calcium (Ca) metabolism are discreetly complementary, and clinically neither can be solely regarded [42]. However, effects of elevated soluble Pi, apparently independent of calcium, have also been demonstrated in vitro and in vivo, for example, direct effects on parathyroid and endothelial cell (EC) dysfunction [43]. Hyperphosphatemia is, therefore, the main hallmark of CKD-related bone-mineral disorders.

### Hyperphosphatemia and Vascular Calcifications in CKD

Disturbed mineral and bone metabolism in patients with CKD, known as CKD–MBD (chronic kidney disease–mineral and bone disorder), leads to vascular calcification (VC) development. Hyperphosphatemia is recognized as a risk factor for VC, as calcium–phosphate deposits in the soft tissues result in higher cardiovascular and cerebrovascular risk in these patients. A high prevalence of intracranial artery calcification in patients with stroke is associated with the presence of CKD [44,45]. It is reported that hyperphosphatemia in dialysis patients increases the risk of a brain hemorrhage [46]. In addition, hypercalcemia in the picture of hyperparathyroidism is linked with enhanced brain calcium levels resulting in neuronal excitability [5]. It is important to note that arterial calcification may affect the large and small arteries in the brain and lead to stroke and cognitive decline, respectively [47]. Indeed, in patients with CKD, both types of VC are present: intimal calcification (atherosclerosis) and medial calcification (arteriosclerosis). Intimal calcification development is associated with higher bone matrix regulatory protein activity and the upregulation of transcription factors: bone morphogenic protein 2, osteopontin, osteocalcin, and runt-related transcription factor 2 (RunX2) [48]. Similarly, medial calcification is associated with bone morphogenic protein 2, RunX2, bone alkaline phosphatase, and Msh Homeobox2 (MSX2), with the final result of the transformation of vascular smooth muscle cells (VSMC) into osteoblast-like cells [48]. In addition, hyperphosphatemia induces VSMCs apoptosis, and calcium phosphate mineralization occurs at these spots, thus enhancing the calcification process [48,49,50,51].

In vitro studies have indicated that culturing human smooth muscle cells in a medium with 1.4 mM phosphate does not induce calcification, but increasing the phosphate concentration from 1.6 to 3.0 mM induced VSMC calcification in a dose- and time-dependent manner [52]. Experimental studies in mice indicated that high phosphate levels induce a vasoconstrictor effect on aortic rings and decrease endothelium integrity and endothelium-dependent relaxation [53]. Sevelamer-HCl treatment improves endothelial dysfunction in CKD mice [53] Moreover, high phosphate levels induce VSMC calcification and increase the expression of the bone-specific transcription factors [54]. Thus, VSMCs change their phenotype toward chondrocyte or osteoblast-like cells [55].

## 4. Phosphate, CKD–MBD Axis, and Neurovascular Dysfunction

High phosphate levels have been associated with endothelial dysfunction and atherosclerosis in CKD patients, not in dialysis [56]. Phosphate excess was also accused as being a main reason for promoting lipid infiltration and atherogenesis [57]. Endothelial cell injury is figured out as the first step leading to atherogenesis, where the endothelial nitric oxide synthase (eNOS) plays an essential role in promoting endothelial dysfunction. Besides, the production of eNOS was significantly reduced under continuous exposure to a high phosphate environment [58]. 

Endothelial cells (ECs), similar to smooth muscle cells SMCs, are one of the major cell types that make up the vessel wall, and their role is crucial in microvascular injury. ECs possess an essential role in VC because they are primary sensors of circulating pathological triggers. Communication through multiple biomolecules and signaling pathways between these two kinds of cells is crucial, especially in VC associated with CKD.

Many studies have gradually found that ECs also have the potential to undergo osteochondrogenic differentiation to promote vascular calcifications [59]. The expression of ossification-related genes in ECs was previously significantly altered under atherogenic and pro-inflammatory stimuli in vitro [60]. A couple of studies showed that ECs could undergo a phenotypic switch and act as a source of bone progenitor cells to promote calcification [61,62]. In addition, more convincing evidence has pointed to the endothelial origin of chondrocytes and osteoblasts. Notably, endothelial–mesenchymal transition (EndMT) provides a reasonable basis for ECs’ phenotypic switch and their osteogenic potential [63]. 

In vitro evidence indicates that high Pi can alter EC morphology and the expression of cell markers and induce EC apoptosis. This process is necessary for EndMT, characterized by the decrease in the expression of endothelial markers (VE-cadherin) and increases in the expression of interstitial markers (S100A4) and the main transcription factors of EndMT (SNAIL, SLUG, and TWIST) [64]. In this way, EndMT allows ECs to develop osteochondrogenic potential, where apoptosis and EndMT in ECs mediated by Pi may further enable VC in CKD [31]. Indeed, high-serum Pi, Ca, and Ca × Pi products are major risk factors for VC. In addition, it was shown that elevated phosphate levels over the physiologic range directly impact endothelial function and vascular remodeling, diminishing microvascular function, angiogenic ability, facilitating endothelial stiffness, and loss of the endothelial ability to repair the protection mechanisms [65].

Apart from the role of Pi in the osteogenic differentiation of VSMCs, it was shown that hyperphosphatemia could induce secretion of IL-8 by EC, which exacerbates the calcification of VSMCs by preventing the production of Osteopontin (OPN). In addition, upon Pi exposure, an up-regulation of the NALP3 inflammasome complex and IL-1β release was observed, where IL-1β encourages the senescence and osteogenic trans-differentiation of VSMCs. In addition, the pro-inflammatory mediators associated with vascular calcification include IL-24 or IL-18 [66]. Furthermore, evidence shows that upregulated FGF-23 expression is a mediator linking elevated serum Pi with inflammation. Other inflammatory biomarkers that raise FGF-23 expression include TNF-α, transforming growth factor-β2 (TGF-β2), and nuclear factor kappa-light chain-enhancer of activated B cells (NFкB), which is an inflammatory transcription factor.

In hemodialysis patients, higher serum phosphate levels were found to be independently linked with an enhanced number of endothelial microparticles (EMPs) and circulating (detached) endothelial cells [67]. Emitted by the endothelium, these circulating submicron-sized membranous vesicles have a significant biological role in vascular injury; EMPs have been shown to act as primary and secondary messengers of vascular inflammation, vasomotor response, thrombosis, angiogenesis, and endothelial survival [68].

The two known type-III NaPiTs—PiT-1 and PiT-2—are involved in phosphate homeostasis in the human body, significantly contributing to the cellular uptake, possibly related to the endothelial dysfunction caused by hyperphosphatemia in patients with CKD. While PiT1 augments vascular calcification through Pi transport-dependent and -independent roles, PiT2 owns a protective role during phosphate exposure [69]. Increased extracellular Pi concentrations are followed by an enhanced intracellular Pi in ECs via active Na+-linked PiT1 (SLC20A1) Pi transporters, enabling Pi to act on intracellular phosphatases and subsequently inducing an intense increase in both protein Tyr and Ser/Thr phosphorylation, are linked with functionally important cytoskeletal rearrangements and the output of EMPs [70]. 

It was recently shown that the direct inhibition by Pi of the phosphoprotein phosphatase PP2A fulfills the sensing role for Pi in ECs. PP2A/Src acts as a potent sensor and amplifier of Pi signals, directly sensing high intracellular Pi where the direct inhibition of PP2A-C by Pi is, therefore, amplified by the feedback loop between PP2A-C and p-Src, resulting in further PP2A-C inhibition, culminating with the disruption of the cytoskeleton and the generation of procoagulant EMPs [71]. 

EMPs are procoagulant and may contribute to clinically meaningful thrombotic events. Procoagulant EMP has also been shown to play a role in atherosclerotic plaques [72,73]. It was shown in rat models that hyperphosphatemia (or a Pi-dependent hormonal comeback derived from it) is sufficient to push a substantial increase in circulating procoagulant EMPs, presenting an essential relation between hyperphosphatemia and thrombotic risk in CKD. Hyperphosphatemia would be clearly portrayed as a biochemical target for future steps to reduce thrombotic disease in CKD due to the defined role of driving accumulations of procoagulant endothelial participles [71]. 

On the other hand, the endothelium produces vasoactive compounds, regulating and stabilizing hemostasis. In uremic conditions, the bioavailability and reduction in endothelial cells may affect this process. One of the essential vasodilator compounds is eNOS that inhibits platelet adhesion and aggregation. In vitro studies have shown that apart from IS, inorganic phosphate decreases NO bioavailability, which is connected, at least in part, to the enhanced oxidative stress in endothelial cells. Endothelial stiffness reflects the adaptations in the structural and functional properties of the endothelium, including restructuring of the cytoskeleton following mechano-signaling activity, intensified endothelial turnover (apoptosis), and diminished NO bioavailability [74]. 

The interaction between ECs and SMCs is widely acknowledged in regulating vessel tone. Vascular stiffness is closely connected to VC, especially with medial calcification. Besides, due to the mechanical stress, ECs can affect SMCs and regulate vessel tone through vasodilators (e.g., NO) and vasoconstrictors (e.g., endothelin1). 

In patients portrayed with endothelial dysfunction, circulating EMPs are inversely linked with the amplitude of flow-mediated dilatation, independently of age and pressure [75]. The EMP levels were associated with lesion volume and clinical outcomes in patients with acute ischemic stroke. Data suggests that EMP levels would be a helpful marker in future risk stratification of patients with a high risk of developing vascular disease. 

Additionally, it would be said that promoting angiogenic processes by EMP may have both advantageous and deleterious effects. EMP could be endogenous survival signals accountable for vascular repair in ischemic tissues. Regardless, the promotion of angiogenic response may also have deleterious effects on atherosclerotic plaque destabilization by enabling intraplaque neovascularization [76]. An underlined factor is elastin remodeling, which plays an essential role in inducing and/or exacerbating SMC phenotype changes. It was shown that the elastin degradation accelerates phosphate-mediated VSMC transformation in rat VSMC culture [77]. 

In CKD and hyperphosphatemia, the FGF23/αKlotho axis is linked to uremic vasculopathy. Hu MC et al. suggested the concept of the ‘endothelial–vascular smooth muscle complex’ should be viewed as potential target of αKlotho in VC in CKD, highlighting crosstalk between the two types of cells [78]. An informative meta-analysis including ten prospective studies demonstrated a significant connection between CKD and cognitive impairment [79]. Neurovascular unit dysfunction associated with multiple structural and functional cerebro-microvascular anomalies is associated with vascular dementia [79]. Besides, the mechanisms offered to explain the possible relations between the elevated PTH found in CKD patients with high Pi, cognitive damage, and dementia are associated with the role of PTH in the regulation of intra- and extracellular calcium, diminishing local brain blood flow and vascular calcifications [80,81]. 

Besides, CKD patients were found to have an increased rate of leukoaraiosis. This pathology of the white matter is linked with perfusion abnormalities in the arterioles that perforate throughout the deep brain structures and is supposed to portray ischemia linked with an elevated risk of stroke and dementia [82]. 

**Figure 1:**
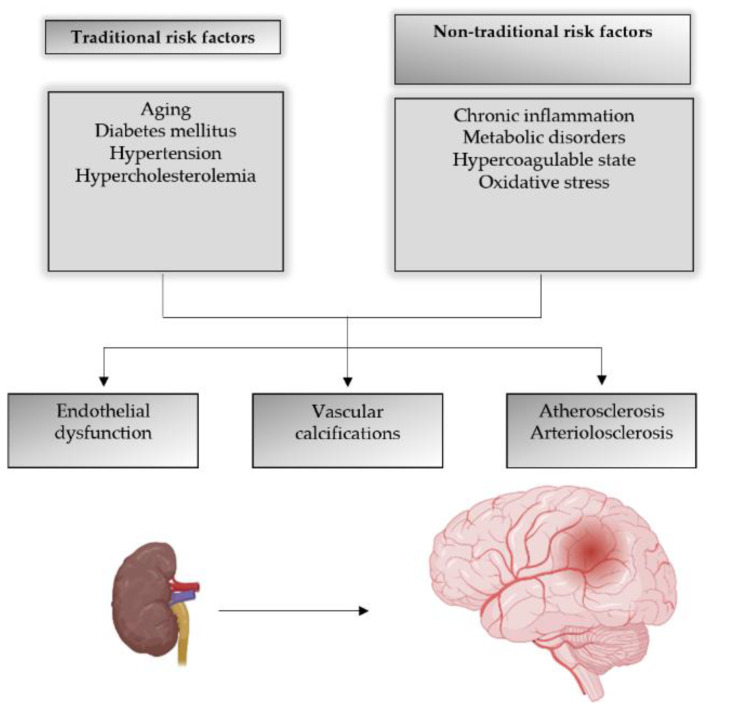
CKD as a risk factor for cerebrovascular disease.

Based on the neuropathological data, concomitant vascular and degenerative brain injury grows the risk of dementia by more than two-fold [83]. Thus, there is a strong chance that CKD patients are at an increased risk for cognitive decline represented by vascular-associated causes, which are defined as brain microinfarcts and white matter disease, and not overt AD per se (Figure 1). In this way, the cerebral vascular condition plays concurrently with a neurodegenerative mechanism partially enabled by uremic toxins.

## 5. Phosphate Impact on Brain beyond the CKD–MBD Axis

A part of increased cardiovascular mortality in CKD patients is significantly attributed to the inflammatory influences of hyperphosphatemia. In addition, CKD has noted an increased vascular inflammation in the absence of atherosclerotic disease [84]. Higher phosphate levels were related to inflammatory markers such as C-reactive protein (CRP) and IL-6. Moreover, a higher phosphate diet was associated with enhanced TNF-α levels and features of oxidative stress. A higher TNF-α is reported to impair synapse function and memory [85]. In CKD patients, low Fetuin-A level patients negatively correlated with CRP levels, which was associated with an increased risk of ectopic calcifications and cardiovascular mortality in hemodialysis patients [86]. Increased expression of FGF-23 in skeletal osteoblasts and osteocytes that downregulates excessive serum Pi levels, is reported to be enabled by hypoxia-inducible factor-1α (HIF-1α) in reaction to inflammation, suggesting that upregulated FGF-23 expression serves as a mediator connecting elevated serum Pi with inflammation. Apart from that, other inflammatory markers such as TNF-α, transforming growth factor-β2 (TGF-β2), and nuclear factor-kappa-light chain-enhancer of activated B cells (NFкB) as an inflammatory transcription factor, are found to increase FGF-23 expression [66,87,88]. 

Research findings point out that phosphate toxicity is a critical biochemical stressor related to the activation of cellular stress response systems. Under circumstances of excess phosphate, an integrated multiple cellular systems result in the activation of autophagy, disintegration of cellular structures in the cytosol by the action of lysosomes associated with increased autophagy, apoptosis, and necrosis [89]. It is biologically conceivable that adrenocortical cell apoptosis is also linked with phosphate toxicity, but additional investigation is needed.

Increased phosphate concentration stresses cellular organelles and promotes the release of reactive oxygen species by mitochondria. Furthermore, hyperphosphatemia is associated with damaged protein tucking in endoplasmic reticulum stress, which may cause an additional inflammatory response, being a mediator between phosphate toxicity and inflammation [90].

### 5.1. The Impact of Hyperphosphatemia on Neurological Disorders

Dysregulation in Pi levels and diurnal cortisol rhythms suggests a relationship between cortisol reaction and phosphate toxicity. An elevated cortisol slope in the morning revealed hypercortisol secretion to be associated with poor health behaviors [91]. Cortisol levels associated with the hyperactivity or hypoactivity of the hypothalamic–pituitary–adrenal (HPA) axis and high serum levels of pro-inflammatory cytokines, are recognized in the etiology of depression, intensely linking depression to stress and inflammation.

Evidence has shown that phosphate toxicity due to hyperphosphatemia may damage the mitochondrial dysfunction in the adrenal cortex and is associated with harmful effects such as calcification and apoptosis. Thus, a novel concept tries to explain the phosphate toxicity as a common reason that dysregulates normal cortisol secretion in hyper- and hypocortisolism [83].

Phosphate toxicity drives chronic inflammation as a trigger of an anti-inflammatory response from the adrenal glands, directing patients towards hypercortisol secretion. A novel concept explains phosphate toxicity as a common reason that dysregulates normal cortisol secretion in hyper- and hypocortisolism, considered as reciprocal interaction between phosphate toxicity and adrenal gland cortisol secretion. Both, hyper- and hypocortisolism are associated with depression features [92]. While hypercortisolism is linked with major melancholic depression, hypocortisolism was observed in patients with chronic fatigue syndrome and comorbid depression, often along with the activation of the sympathetic nervous system. Of note, the inflammation rather than cortisol level is related to the depressive fatigue. Depression is associated with anomalies of FGF23, which in turn controls phosphate homeostasis through phosphaturia [93]. Phosphate toxicity is also proposed to impair adrenal function via tumorigenesis, ectopic calcification, and adrenal cell apoptosis, necrosis and autophagy, leading to hypocortisol secretion.

The proposed mechanism is based on the upregulation of the biogenesis of ribosomal RNA by inorganic phosphate, which increases cellular protein synthesis, and encourages tumor growth [94]. Continuous activation of nNOS leads to an increased production of NO and reactive oxygen and nitrogen species, inflammation, and mitochondrial dysfunctions, which are the pathophysiological bases of many neurological complications in CKD. Found in specific brain regions of the mice, they are considered underlying reasons for the observed neurochemical and histopathological alterations [95].

Interestingly a murine model of neurological complications in CKD demonstrated mitochondrial dysfunction, oxidative stress, and inflammation in the mouse brain. Thus, it is hypothesized that these factors might interfere with the synthesis, release, and uptake of dopamine in the basal ganglia circuitry of the brain of CKD mice and the resulting motor abnormalities. Additionally, hyperphosphatemia may also influence dopamine metabolisms, as well as uremic factors such as anemia, iron, and erythropoietin deficiency [95].

Apart from vascular dementia, higher levels of serum Pi were associated with the enhanced risk of Alzheimer’s disease and Lewy body dementia [15]. The relation of altered levels of phosphate in the cerebral spinal fluid has been noted to be associated with different neurologic pathologies such as epilepsy, inflammatory diseases associated with disorders in the blood–brain barrier, idiopathic basal ganglia calcification, delirium following hip fracture, and tumors of the CNS [30]. In addition, serum levels of Pi were also significantly associated with cognitive decline in a 2-year cohort study of elderly outpatients with pre-dialysis CKD [96]. The general perception is that accumulation of amyloid plaque in the brain is linked to Alzheimer’s disease. Recently, it was reported that functional decline starts in the brain before amyloid plaque accumulation occurs, connecting cognitive impairment with other features such as the deposition of intracellular neurofibrillary tangles (NFTs) formed by hyperphosphorylated tau protein. Tau is a unique protein that plays a crucial role in cognitive processes. However, deposits of abnormal forms of tau are aggregated intracellularly in the neurons, leading to neuronal damage and autophagy and mitochondrial dysfunction, synaptic deficits, and neuronal death, which eventually lead to neurodegeneration and cognitive decline, including AD [97]. The neurodegeneration associated with abnormally hyperphosphorylated tau found in Alzheimer’s disease was linked with abnormal phosphate levels (between 20 and 40 mM). In addition, self-assembled NFTs occur as negative charges added to hyperphosphorylated tau, leading to molecular binding with normal tau [98]. 

Another clue is that neuroinflammation of the brain also plays an integral part in the pathogenesis of AD. Pro-inflammatory cytokines, including interleukin-1 (IL-1), IL-1β, IL-6, IL-18, TNF-α, and interferon-γ (IFN-γ), shift to an abnormal phosphorylation of tau by stimulating tau kinases, such as cyclin-dependent kinase 5, p38 mitogen-activated protein kinases, and glycogen synthase kinase-3β. There is supportive evidence that neuroinflammation, brain cell shrinkage, and apoptosis in AD could be related to the damaging cellular effects of phosphate toxicity [30].

It is reported that 90% of cell death occurs when phosphate concentrations reached 64 mM apoptosis, while cell shrinkage and nuclei fragmentation occurred over 40 mM of Pi level [32,98]. 

The mechanisms offered to explain the possible relation between elevated PTH found in CKD patients with high Pi, cognitive damage, and AD is linked with the role of PTH in the regulation of intra- and extracellular calcium and diminishing local brain blood flow [99]. Moreover, it is thought that associated calcium may induce neuronal signaling disruption [100]. Recently, a significant increase in gray matter volume (GMV) in ESRD patients with secondary hyperparathyroidism has been reported to be associated with cognitive impairment [101]. Data show that the short-term improvement in depression and anxiety after medical normalization of hypercalcemia is also associated with long-term cognitive function effects six months after parathyroidectomy [102]. Notably, a recent study of 189,433 older adults without a diagnosis of dementia showed that the treatment of secondary hyperparathyroidism (vitamin D analogs, phosphate binders, calcimimetics, or parathyroidectomy) was associated with a 42% lower risk of incident dementia and 33% of AD among older patients (age ≥ 65) with ESRD [103]. Similarly, the presence of FGF-23 in the brain along with the increased levels of FGF23 correlated with memory decline, as estimated by composite scores [104]. Additionally, vitamin D is a well-known factor in regulating calcium homeostasis, β-amyloid deposition, antioxidant, anti-inflammatory properties, and neuroprotection against neurodegenerative processes linked with dementia, Alzheimer’s disease and cognition. Low levels of vitamin D were linked with the development of dementia and depression. In CKD, where low vitamin D is observed, we could attribute a part of cognitive disorders to vitamin D deficiency [105]. 

**Figure 2:**
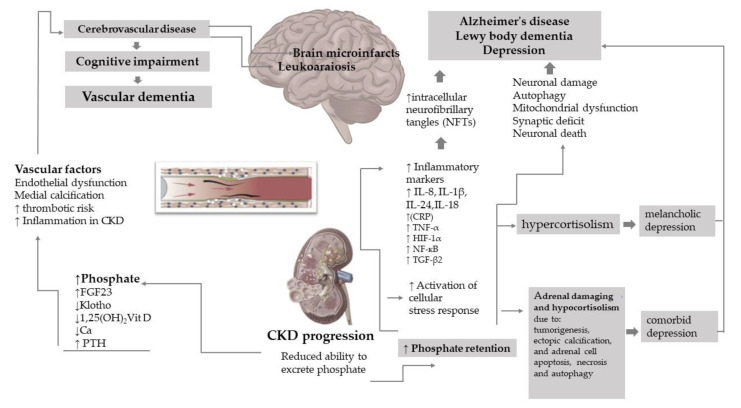
The impact of phosphate toxicity in the brain. Abbreviations: FGF23—fibroblast growth factor 23; PTH—parathyroid hormone, Ca—calcium; PTH hyperparathyroidism; Vit D—vitamin D; (IL-8)-Interleukin; (IL-1β)-Interleukin-1β; (IL-18)-Interleukin-18; (IL-24)-Interleukin-24; (CRP)—C Reactive protein; (TNF-α)-tumor necrosis factor alpha, (HIF-1α)-hypoxia-inducible factor-1α, (NFкB)-nuclear factor kappa-light chain-enhancer of activated B cells, (TGF-β2) Transforming growth factor-β2; ↑—increased; ↓—decreased.

Therefore, hyperphosphatemia and other metabolic disorders linked to CKD can significantly impair the CNS (Table 1) (Figure 2). This consequence cannot be underestimated, considering the higher morbidity/mortality, lower quality of life, and associated high health care costs for neurological disorders [30]. 

### 5.2. The Impact of Hypophosphatemia on Neurological Disorders

Finally, attention is also needed to be paid to hypophosphatemia, a risk factor for encephalopathy development. Clinical cases of hypophosphatemia-induced encephalopathy after hemodialysis sessions have resolved after serum phosphate correction [106,107]. Likewise, hypophosphatemic encephalopathy is possible in continuous ambulatory peritoneal dialysis patients [108].

A study on hypophosphatemia after nontraumatic intracranial hemorrhage showed that acute hydrocephalus and diffuse brain edema were more common in patients with hypophosphatemia; however, there was no difference in outcome between hypophosphatemic and normophosphatemic patients [109]. Hypophosphatemia can also cause leukoencephalopathy, which can be corrected with treatment [110]. Other neurological illnesses, such as osmotic demyelination syndrome, have been described to be also associated with hypophosphatemia [111]. 

The latest data open a new window for the control of phosphate levels within and beyond the context of CKD-MBD, raising the question of whether the control of hyperphosphatemia and related biomarkers in CKD would lower patients’ risk of developing cognitive impairment and dementia. In this regard, future research is needed, and maybe this could be associated with new targets that could modify the risk. 

The proposed mechanism that connects hyperphosphatemia in the context of CKD-MBD with cerebrovascular disease is on the left side of the figure. Neurovascular unit dysfunction was linked with multiple structural and functional cerebro-microvascular anomalies that diminish local brain blood flow and are associated with vascular dementia. In addition, CKD patients were found to have an increased rate of leukoaraiosis. This pathology of the white matter is linked with perfusion abnormalities in the arterioles that perforate throughout the deep brain structures and is supposed to portray ischemia linked with an elevated risk of stroke and dementia. Thus, there is a strong chance that CKD patients are at an increased risk for cognitive decline represented by vascular-associated causes, defined as brain microinfarcts and white matter disease. On the right side is presented the role of phosphate toxicity beyond CKD–MBD. A part is attributed to the increased risk of systemic inflammation and cellular stress response. Both mechanisms impact cortisol levels and increase the risk of depression. Evidence has shown that phosphate toxicity due to hyperphosphatemia may damage the mitochondrial dysfunction in the adrenal cortex and is associated with harmful effects such as calcification and apoptosis. Apart from vascular dementia, higher levels of serum Pi were associated with the enhanced risk of Alzheimer’s disease and Lewy body dementia. It was reported that functional decline starts in the brain before amyloid plaque accumulation occurs, connecting cognitive impairment with other features like the deposition of intracellular neurofibrillary tangles (NFTs) formed by hyperphosphorylated tau protein. Another clue is that neuroinflammation of the brain also plays an integral part in the pathogenesis of AD. There is supportive evidence that neuroinflammation, brain cell shrinkage, and apoptosis in AD could be related to the damaging cellular effects of phosphate toxicity.

## 6. Conclusions

In summary, current evidence from basic research suggests the paramount role of phosphate in developing neurological disorders in the frame of CKD. However, clinical data investigating this relevant topic are limited. Therefore, it would be necessary to further evaluate the link between serum phosphate and cognitive impairment (and other neurological disorders) in the clinical setting. Overall, it is important to define what is the balance between phosphate intake, when phosphate is “good for the brain”, and when it starts to be harmful, as seen in chronic kidney disease patients. Moreover, it would be also interesting to assess the potential improvement of neurological disorders in CKD in well controlled hyperphosphatemia, and eventual improvement after renal transplantation. Drugs that reduce phosphate levels (phosphate binders, calcimimetics) might be proven as beneficial for the brain, which should be studied in further subsequent studies.

## Figures and Tables

**Table 1:** CKD–MBD biomarkers, role in bone metabolism, vascular calcification, brain, and neurocognitive function.

CKD-MBD Biomarkers	Role in Bone Metabolism	Vascular Calcification	Brain and Neurocognitive Function
PTH	Key mediator of bone turnover Regulates P and Ca homeostasis	Complex paracrine and systemic effect Promotes VC Impairs endothelial function	Endothelial dysfunction Arterial stiffness ↑Ca→ neuronal signaling disruption, frontal-subcortical dementia, atrophy in hippocampus ↑PTH→significantly decreased gray matter volume (GMV) Impair executive function memory impairment
Vit D	Key role in Ca, P homeostasis Depletion promote sHPTH and osteitis fibrosis cystica	Biphasic curve of Vit D on calcification	↓Vit D→ impairs contractile function of vessels ↓Vit D→ may lead to disruption of calcium homeostasis in neurons, thus causing neuronal aging and neurodegeneration vulnerability ↓Vit D→↑Amyloid β deposition and tau protein tangles in the brain ↓Vit D→↑inflammation, ↑oxidative stress, endothelial dysfunction
Klotho	Acts as a Wnt-inhibitor Modify bone metabolism	Inhibitor of VC Klotho deficiency→ impair endothelial function	Klotho deficiency →impairment of vascular cell senescence, →impairment of brain immune system, →impairment of central nervous system
FGF23	Posphaturic hormone acts through α-klotho	Is not clear if it has a direct effect on VC	↑FGF23 not directly connected with cognitive impairment ↑FGF23→↓Vit D ↑FGF23→↑inflammation

Abbreviations: CKD-MBD—chronic kidney disease—mineral and bone disorder; PTH—parathyroid hormone; P—phosphate; Ca—calcium; VC—vascular calcification; sHPTH—secondary hyperparathyroidism; Vit D—vitamin D; FGF23—fibroblast growth factor 23; Wnt—wingless-related integration site; ↑—increased; ↓—decreased; →—linked to.

## Data Availability

Not applicable.
